# Environmental Factors as the Main Hormonal Disruptors of Male Fertility

**DOI:** 10.3390/jcm13071986

**Published:** 2024-03-29

**Authors:** Natalia Wdowiak, Kamila Wójtowicz, Anita Wdowiak-Filip, Weronika Pucek, Andrzej Wróbel, Jan Wróbel, Artur Wdowiak

**Affiliations:** 1 Chair of Obstetrics and Gynecology, Faculty of Health Sciences, Medical University of Lublin, Staszica 4-6 Street, 20-081 Lublin, Poland; natalia.wdowiak62@gmail.com; 2 Department of Gynecology and Obstetrics. Municipal Hospital, Saint Michael the Archangel in Łańcut, Parens, Infertility Clinic in Rzeszów, 35-309 Rzeszów, Poland; guccii@poczta.onet.pl; 3 Department of Cosmetology and Aesthetic Medicine, Medical University of Lublin, Chodzki 1, 20-093 Lublin, Poland; anita.wdowiak@gmail.com; 4 National Medical Institute of the Ministry of the Interior and Administration, 02-507 Warsaw, Poland; weronika.pucek@gmail.com; 5 Second Department of Gynecology, Medical University of Lublin, Jaczewskiego 8, 20-090 Lublin, Poland; wrobelandrzej@yahoo.com; 6 Medical Faculty, Medical University of Lublin, 20-093 Lublin, Poland; wrobeljan@onet.eu

**Keywords:** male fertility, semen, environmental factors, oxidative stress, infertility

## Abstract

Introduction and objective: Many scientific reports confirm a systematic decline in male semen parameters over the last decades. This phenomenon has been observed in all parts of the world, and its occurrence is associated, among others, with the hazardous effects of some environmental factors. The environmental factors for which the adverse effect on male fertility has been proven include water, air, and soil pollution, as well as electromagnetic fields and ionizing radiation. The aim of this article was the evaluation of the effect of selected environmental factors on male reproductive capacity based on an analysis of the current scientific reports. Review methods: A systematic literature review was carried out using three databases: PubMed, EMBASE, and Scopus. The search was limited to the period from 2015 until the end of December 2023. Brief description of the state of knowledge: Environmental factors, such as heavy metals, tobacco smoke, pesticides, dioxins, furans, phthalates, and bisphenols, are well-tested substances that exert an adverse effect on male fertility. A harmful effect of electromagnetic fields and water and air pollution on reproductive functions may be expected; however, this has not been fully proven. Summary: Results obtained by many researchers published to date should evoke great concern regarding the quality of the environment in which we live, as well as fears about the effect of environmental factors not only on male fertility, but also on all aspects of human health. The majority of environmental pollutants affect the male body by causing oxidative stress and through their effect on the endocrine system.

## 1. Introduction and Objective

Infertility is defined as the failure to achieve pregnancy within a year of attempting to become pregnant [1]. Almost every sixth couple in Europe experiences problems with obtaining offspring. It is considered that a male factor is responsible for almost half of the cases of problems conceiving offspring [2]. Male fertility is determined primarily based on the examination of semen. Many scientific reports confirm a systematic decline in the parameters of male semen over the last decades [3,4]. This phenomenon is observed in all parts of the world, and its occurrence is associated, among others, with the adverse effect of some environmental factors [5]. The environmental factors for which the unfavorable effect on male fertility has been proven include water, air, and soil pollution, as well as electromagnetic fields and ionizing radiation [6]. The harmful impact of these factors on male fertility is most frequently explained by the concept of oxidative stress, as well as their effect on the secretion of sex hormones [7]. Harmful factors may affect the male body at all stages of life, starting as early as the fetal stage [8,9,10].

Experts sound the alarm that population decline and not the depletion of resources may end the steady growth of per capita income and consumption. Population growth must keep pace with the growth in consumption and income. Rising profits alone cannot maintain sustained growth in per capita income and consumption, and population expansion is necessary for sustainable economic growth with exhaustible resources [11].

In the years 1970–2020, the fertility rate decreased in all countries worldwide. Although the world’s population continues to grow, the rate of this growth is decreasing. As a matter of fact, this has been happening since the 1960s. Apparently, the situation differs depending on the country. Demographic growth remains considerable in poor African countries, and the opposite trend is observed in wealthy European countries. Nevertheless, the global situation leaves no doubts. Population aging is the omnipresent global demographic trend, due to decreasing fertility, increasing longevity, and the progression of large cohorts into older age [12]. This phenomenon is accompanied by a decrease in male fertility. Among unselected men from all continents, the mean sperm concentration declined by 51.6% between 1973 and 2018 [13].

This also means colossal economic, health, and social challenges in the coming decades. If these challenges are not met, then the consequences will be extremely serious, from the increase in age-related diseases, through an excessive burden on healthcare and pension systems, to the declining quality of life of older people resulting from the overloading of wage earners.

The aim of this article was the evaluation of the effect of the selected environmental factors on male reproductive capacity, based on an analysis of the current scientific reports.

## 2. Review Methods

A systematic literature review was carried out using three databases: PubMed, EMBASE, and Scopus. The search was limited to the period from 2015 until the end of December 2023. The following approach to the literature search was applied using proper keywords: male fertility, semen, environmental factors, oxidative stress, and infertility. On this basis, the environmental factors presented in individual chapters were selected. The inclusion criteria for articles were as follows: English language of the manuscript, study type—systematic review or observation study (cohort, case–control, or cross-sectional), and studies of environmental risk factors. The exclusion criteria were as follows: non-English language, study type—non-systematic review articles, case reports, and studies of non-environmental risk factors.

## 3. Interactions between Environmental Factors and Organisms

Each organism possesses an individual capability for adaptation to various environmental factors, defined as ecological tolerance. The principle of operation of environmental factors has been described by the Liebig’s Law of the Minimum and the Shelford’s Law of Tolerance in ecology. The first law assumes that the scarcest factor has a limiting effect on an organism or on the whole population. In turn, the second law proves that both excessively low and excessively high intensities of various environmental factors have a limiting effect on organisms. According to this law, an organism may function between the minimum and maximum effect of the factor, i.e., within the range of tolerance [14].

Harmful environmental factors related to inanimate nature exert an impact on a living organism, the effect of which depends on the duration of action, dose, and genetically conditioned individual defense capabilities of an organism. Individual responses to environmental factors result from the presence of genetic polymorphisms responsible for the metabolism of enzymatic processes that counteract the harmful influence of select environmental factors. Human inter-individual variability depends on age, weight, ethnic origin, gender, concomitant diseases and co-exposures, as well as medications taken [15]. An example of this is research that has proven that Metformin administration provided protection against oxandrolone-induced infertility in male rats [16].

Σ (dose/genome) × time = spermatogenesis disorders

Male reproductive capacity is shaped already during the pre-conception period of a given individual. Environmental factors affecting the gametes of the mother and the father may determine the fertility of their son. It is similar during the fetal period, an example of which is phthalate syndrome. The harmful effect of some environmental factors may have an impact on male fertility throughout their whole life; however, most substances undergo biotransformation, except for highly polar compounds (e.g., phthalic acid) and volatile compounds (e.g., ethyl ether). Unmetabolized substances may bioaccumulate in the fatty tissue in the liver (e.g., organochlorine pesticides, dioxins), as well as in the kidneys, bones, and liver (some heavy metals) [17,18].

The harmful effect of environmental factors on male fertility is explained primarily by the concept of oxidative stress. Reactive oxygen species are physiological products of tissue metabolism and, secreted in appropriate amounts, play an important role in many life processes. Their effect is regulated by antioxidant systems of the body. In the case of increased production of reactive oxygen species, the distortion of the balance between their production and the action of the protective antioxidant system results in the occurrence of oxidative stress [19]. This phenomenon leads to damage to cell organelles, disturbances to their function, and subsequently to cell apoptosis. It is suspected that disorders in the free radical system are among the major causes of male infertility, including idiopathic infertility [20]. Due to the popularization of measurements of the oxidation–reduction potential of semen, it is possible to diagnose male infertility caused by oxidative stress (Male Oxidative Stress Infertility—MOSI) [21].

In addition to oxidative stress, the harmful effects of environmental factors are related to their impact on epigenetic processes. Epigenetic modifications refer to heritable changes in the function of a gene, which take place without changing the nucleotide sequence of the DNA. Epigenetics may be seen as an essential ‘biostat’ which, in response to environmental changes, enables a tissue or organism to turn on and off the anticipatory programs of gene transcription, which results in adaptive phenotypic alterations for better survival. The programs of gene transcription change in response to environmental conditions immediately or over a long period of time. As a result of the methylation of DNA, modification of histones, transcription of new micro- and long non-coding RNAs, and remodeling of higher-order chromatin structure, new adaptive traits are developed. A class of proteins called ‘chromatin modifying enzymes’, which are extremely sensitive to particular environmental changes, generates, maintains, and removes these epigenetic modifications. On the contrary, heritable or sporadic epimutations, or disruptions of the epigenome in a tissue under harmful environmental conditions, may lead to the development of a disease [22].

Genetic susceptibility is a genetic characteristic predisposing organisms to the effect of irritating environmental factors, such as chemicals, air pollution, heavy metals, radiation, or occupational dust. A growing interest is observed concerning the involvement of human genome polymorphisms in the modification of the impact of exposure to environmental hazards, making individuals or population groups prone to varying degrees to the development of a disease following exposure. Living things exist due to the occurrence of stable genetic novelties facilitating the adaptation of organisms to harsh environmental conditions. This adaptation results from the interaction between genetic factors and the novel environment; it may be favorable or have adverse effects, leading to the development of a pathological process [23].

Mature sperm is especially susceptible to the effect of environmental factors, because, in practice, it does not possess repair mechanisms protecting it against damage caused by oxidative stress. Damage to the genetic material of sperm may be repaired by the oocyte. However, capabilities for the repair of fragmented DNA depend on the degree of damage and individual properties of the egg cell [24].

Nevertheless, the parameters of male semen do not fully determine male reproductive capacity. Examination according to the WHO standard 2021 cannot guarantee whether the sperm will manage to penetrate the partner’s reproductive tract, where hyperactivation, acrosome reaction, and fertilization occur. A manual assessment of semen is unable to evaluate either genetic material or epigenetic processes in semen, or balance in the oxidation–reduction system. Extended semen diagnostics and experimental tests not commercially available provide additional information concerning reproductive capacity [25]. Many scientific reports confirmed an adverse effect of environmental factors on sperm functions, assessed by microscopic analysis, as well as by extended semen diagnostics [26].

## 4. Heavy Metals—Lead, Cadmium, Mercury

These elements are omnipresent in the environment and accumulate in the human body throughout life, as well as during the prenatal period. Heavy metals have the ability to generate oxidative stress in the tissues of the human body. Lead (Pb) and cadmium (Cd) are well-known heavy metals exerting a harmful effect on male reproductive capacity. The non-ferrous metal mining and processing industry, as well as burning waste, oil, and coal, contribute above all to the contamination of the environment. Large amounts of cadmium also enter the atmosphere during volcanic eruptions. Active and passive smokers are exposed to its toxic effect [27].

The testicles are very sensitive to its effects. Fetal exposure to cadmium is capable of causing disorders of fetal organogenesis, later problems with testicular descent, and impairing their function during reproductive age. Cadmium may contribute to damage to the vascular endothelium and blood vessels in the testicles. It generates oxidative stress causing an inflammatory response and testicular dysfunction. The consequence of this is decreased testosterone secretion due to interstitial edema and necrosis in the seminiferous tubules, which impairs the process of spermatogenesis. In addition, cadmium damages intercellular junctions in Sertoli cells. Moderate exposure to cadmium causes a decline in all main semen parameters, and is the cause of erectile dysfunction [27,28].

The main natural sources of lead emissions into the human environment are volcanic eruptions, the weathering of rocks, and forest fires. This element also enters the environment as a result of industrial human activity in the transport sector, the lead ore mining and processing industry, and as a consequence of the production of batteries, paints, enamels, and varnishes. Exposure to this element results in the reduction in volume of the testicles, epididymis, prostate gland, and seminal vesicles. It also contributes to peritubular fibrosis and the vacuolization of Sertoli cells. Leydig cell dysfunction is caused by decreases in Pb testosterone secretion, and impaired spermatogenesis causes a decrease in semen density and the percentage of sperm with normal structure.

Human exposure to Pb and Cd is often accompanied by exposure to zinc (Zn). Low doses of metals, such as zinc and copper (Cu), have a positive impact on male fertility, eliminating the harmful effect of Cd and Pb. Copper, zinc, and selenium (Se) are indispensable for reproductive health because they are activators of some antioxidant enzymes; however, increased exposure to these elements exerts an unfavorable effect on fertility. Lead, by acting antagonistically or competitively with selenium, copper, and zinc, may additionally disturb the antioxidant defense of cells [27,28].

Mercury (Hg), commonly present in the human environment, is a heavy metal with very strong toxicity. The risk of contact with mercury occurs especially among persons employed in the mining industry, chemical plants, and in the production of dyes and plant protection products. Considerable amounts of this element are found in some marine and freshwater fish populations intended for consumption. Mercury disrupts the biosynthesis of proteins through the reaction with -SH enzyme groups, which inhibits their activity and also causes changes in DNA phosphate bonds. Mercury disrupts the function of the blood–testis barrier, exerting a negative effect on all main semen parameters [27,28].

## 5. Air Pollution

The Organization for Economic Co-operation and Development declares that by the year 2050, air pollution in cities will be the main environmental cause of death worldwide, overtaking deaths caused by the poor quality of drinking water and lack of proper sanitary conditions. In Poland, air pollution is the cause of approximately 45,000 premature deaths annually, and 97% of Poles breathe air that is considered by the World Health Organization (WHO) to be harmful to health. The low quality of the air in Poland is primarily due to the emission of pollutants from the municipal and domestic sector and road transport. The consequence of this is the release of significant amounts of exhaust gases into the atmosphere, including: sulfur oxides (SO_2_, SO_3_), nitrogen oxides (NO_X_), carbon oxides (CO, CO_2_), as well as dust, soot, and ashes. PM10 dust present in the air (particles with a diameter less than 10 μm) and PM2.5 (fine dust particles with a diameter less than 2.5 μm) are very hazardous to health because they contain in their composition lead, sulfur, mercury, nitrogen compounds, and other heavy metals [29,30].

The results of many scientific studies also confirm an unfavorable effect of air pollution on male semen parameters. Some researchers considered exposure to PM2.5 as a factor decreasing sperm motility, whereas others have not observed such a relationship. It was also confirmed that SO_2_ and NO_2_ reduce the reduction in sperm progressive motility. An unfavorable effect of PM10, NO_2_, and SO_2_ on sperm morphology has also been proven by some researchers, while others did not confirm such an effect of these substances. In turn, a relationship was confirmed between exposure to PM2.5 and CO and disorders in sperm morphology. The negative effect of PM2.5 on sperm density was described in several scientific studies. There are also many reports concerning an unfavorable effect of air pollution on the phenomenon of sperm DNA fragmentation; nevertheless, this is not confirmed by all studies. In turn, the effect of PM2.5 on the development of disomy Y.21 was proven [29,30].

## 6. Pesticides

Pesticides are biologically active chemical compounds of natural or artificial origin, applied in agriculture as plant protection products. They include several groups of substances: organochlorine pesticides banned in the European Union (carbon tetrachloride, chlordane DDE, DDT, dieldrin), organophosphate pesticides (chlorpyriphos, malathion, acephate), carbamates (aldikarb, carbaryl, methiocarb, maneb), neonicotinoids (thiamethoxam, clothianidin, imidacloprid), and pyrethroids (cyhalothrin, cypermethrin, deltamethrin, permethrin). Exposure to contact with these substances concerns nearly everyone, because they are present in soil, water, and edible plants. They cause acute or chronic poisonings. Their chemical structure is similar to steroid hormones. Due to this, they have the ability to act antagonistically towards estrogen or androgen receptors. Pesticides may affect the synthesis, metabolism, and transport of hormones. Their harmful effect is also explained by the concept of oxidative stress caused by the production of reactive oxygen species. In males, pesticides cause damage to seminiferous tubules, disorders in hormone secretion, deterioration of sperm motility, and damage to the sperm genetic material [31,32].

## 7. Dioxins and Furans

Dioxins and furans are substances classified into persistent organic pollutants. These chemical compounds include polychlorinated and polybrominated dibenzodioxins and polychlorinated and polybrominated dibenzofurans. They are emitted into the environment as a result of combustion of municipal waste with too low an oxygen content at too low a temperature, especially those containing chlorine and sludge from sewage treatment plants. Dioxins are also emitted by the transport industry and the metallurgical and chemical industry.

Dioxins and furans are characterized by high toxicity and bioaccumulative potential, showing high persistence in our environment. Dioxins, after entering the body via the alimentary route, accumulate in the fatty tissue and the liver. These compounds, similar to pesticides, disrupt the function of the endocrine system. Male gonads are very susceptible to their influence, which can exert harmful effects on male fertility even during fetal development. Dioxins and other substances with biological effects that mimic estrogen (so-called xenoestrogens) in the English literature, described as endocrine-disrupting compounds (EDCs) or endocrine disruptors (EDs), are responsible for causing testicular dysgenesis syndrome (TDS) as a result of effects on the male fetus. Males with TDS have decreased semen parameters and a low level of testosterone as a result of hypergonadotropic hypogonadism; they may develop cryptorchidism, hypospadias, and are at increased risk of developing testicular cancer. The testicular ultrasound examination shows a reduced volume of the testicles, and numerous microcalcifications are visible on the cross-section of the gonad. Despite the occurrence of various types of TDS, histopathologic changes in the structure of the testicles are similar. The sperm-forming tubules are characterized by a reduced diameter and the content of immature Sertoli cells, and sometimes the Sertoli cells themselves, as well as hyaline bodies and calcifications. The intertubular spaces are widened, and Leydig cells are overabundant. In the case of severe forms of TDS, testicles may be located in the abdominal cavity or inguinal canals; in addition, hermaphroditic or female external genitalia may be present despite the presence of the Y chromosome, or part of it in the karyotype; sometimes, there are poorly developed male and female or only female internal genital organs, or a decreased level of testosterone in blood serum and lower testosterone secretion reserve compared to age-mates in the hCG test before and during puberty, as well as already excessive levels of LH and FSH in pre-adolescence and extremely high levels in adults [33,34,35].

## 8. Bisphenol A

Bisphenol A (2,2-Bis(p-hydroxyphenyl)propane—BPA) is classified into the organic chemical compounds from the phenolic group. It is used as an ingredient for manufacturing plastics, due to which it is present nearly everywhere. BPA is included in plastic bottles, food containers, thermal paper for printers and terminals, epoxy resins, paints, and varnishes. It is also used as an antioxidant in the food industry and for the production of cheaper cosmetics.

BPA enters into food and drinks from packaging, especially when the fluid temperature increases, and also as a result of damage to the structure of the plastic packaging (cracks, crushing, washing). Similar to dioxins, BPA belongs to the group of xenobiotics and exerts an effect on the hormonal balance of the body. Exposure to BPA results in the deterioration of the quality of sperm by decreasing the number of sperm, motility, and percentage of normal sperm. There is inconclusive evidence that BPA intensifies sperm DNA fragmentation. Exposure to BPA in childhood may cause precocious puberty [36].

## 9. Phthalates

Phthalates are salts and esters of phthalic acid, which act as plasticizers that give plastics flexibility, and are also components of resins, adhesives, air fresheners, and household chemicals. The use of phthalates, i.e., di(2-ethylhexyl) phthalate and di-n-butyl phthalate, has been banned for the production of cosmetic dyes and toys, and products intended for children. Contact with these substances takes place through the oral route as a result of products contaminated with phthalates during food storage or processing. Additionally, exposure may occur through the skin as a result of contact with cosmetics and by inhalation. Similar to BPA or dioxins, phthalates belong to endocrine disruptors. Their effect has been very well recognized in animal models and described as ‘phthalate syndrome’. In experiments on days from 18–21 of pregnancy, the fetus was exposed to the effect of phthalates, which resulted in abnormalities of the penis and its reduction, undescended testicles, and shortening of the distance between the anus and the penis. Phthalate syndrome described in animal models is identical to the previously described dysgenetic testicular syndrome in humans. In the case of breeding individuals with this syndrome, the resulting offspring do not inherit any abnormalities from the father [37].

## 10. Pharmaceutics and Estrogens in the Aquatic Environment

An important problem for the aquatic environment is micropollutants, including pharmaceuticals and sex hormones. They occur in small amounts and are difficult to remove. Their effect on aquatic ecosystems and the state of human health has not been examined in the long term. Unfortunately, commonly used wastewater treatment methods do not in any way enable the removal of the substances mentioned, or they remove them only to a small extent. Among the trace contaminants detected in water reservoirs are active pharmaceutical compounds, residues of products used for personal hygiene, artificial sweeteners, pesticides, surfactants, chlorinated organic compounds, polycyclic aromatic hydrocarbons, furans, corrosion inhibitors, and generally endocrine disrupting chemicals [38,39].

Micropollutants including pharmaceuticals, especially estrogens, may penetrate into the environment during the removal of untreated sewage from hospitals, pharmaceutical plants, veterinary facilities, discharge of treated sewage from municipal sewage treatment plants, leakage from landfills (expired medications improperly disposed of), or industrial animal breeding. A source of groundwater contamination may also be improperly operated home sewage treatment plants or septic tanks.

It is estimated that the entire world’s human population introduces into the environment approximately 30 tons of natural estrogens and about 1 ton of synthetic compounds annually. According to the Registration, Evaluation and Authorisation of Chemicals (REACH), between 10 and 100 tons of estrone are imported to Europe annually. The European Chemicals Agency publicly informs on the use of this hormone in pharmaceuticals; however, it does not provide a list of products in which it is used. The routes of releasing it into the environment are also unknown [38,39]. Groups of experts dealing with this scope of problem postulate that this situation may be responsible for the risk of the development of dysgenetic testicular syndrome [40].

## 11. Ionizing Radiation

Radioactivity is the ability of atomic nuclei to undergo radioactive decay, which is associated with the emission of alpha particles, beta particles, and gamma radiation. As a result of this process, new atomic nuclei are created. Its severity is determined by indicating the activity of the radioactive source. Radiation accompanying nuclear transformations, passing through the substance of the medium—the surrounding environment—causes ionization involving the removal of electrons from atoms. Ionizing radiation (IR) contains alpha, beta, gamma, UV, and X radiation. Radioactivity is an inseparable component of the human environment. There are radioactive isotopes in our environment of natural and artificial origin, resulting from human activity (mainly cesium and strontium). The problem of exposure to radioactivity of this origin occurs primarily in the mining of hard coal and other minerals, where among others, exposure to radon and derivatives of its decay in mine air, as well as to gamma radiation from natural isotopes, mainly radium, contained in rocks of the rock mass, and water with an increased content of radium isotopes occur. It should be emphasized that crews of planes reaching high flight altitudes of 10,000 m are also among the occupational groups most exposed to radiation. Today, an important issue is exposure to ionizing radiation related to medical diagnostics, concerning both patients and medical staff. In Poland, exposure to radiation concerns tens of thousands of people. In this group, the highest percentage constitutes employees of the medical sector [41,42].

The effect exerted by ionizing radiation on organisms is determined by dose size and the type of radiation. The biological effects of this process also depend on the irradiation conditions, such as dose rate, method of dose fractionation, mass, type, and oxygenation of irradiated tissues (hypoxia protects against damage resulting from irradiation). The basic pathomechanism of cell damage is the result of the formation of free radicals under the influence of ionizing radiation (water radiolysis). In addition, among others, damage to lysosomal membranes and the release of Fe ions into the cytoplasm occur, which increases the amount of DNA damage and cell mortality. This is the so-called indirect effect of radiation, involving the absorption of radiation by a medium and the production of intermediate products damaging macromolecules, the percentage of which in terms of the biological effects of radiation is estimated at 60–70%. The remaining part (30–40%) is a direct effect of the direct deposition of radiation in the molecule. The impact of radiation on DNA is due to both effects and may result in damage to the following DNA components: the sugar skeleton and the nitrogenous bases. The most frequently occurring damage types are oxidative damage to bases, loss of base, strand breaks, and cross-links [41,42].

The effect of radioactivity on spermatogenesis has been best examined in animal models on rodents. The consequences of testicular irradiation in rodents include both macroscopic changes (dose-dependent decreased testicular weight on days 16 and 45 after exposure to the dose from 4 to 1 Gy), and microscopic changes (reduction in cells in the seminiferous tubules and the reduction in the number of sperm occurring on day 45 after exposure to 0.25 Gy). For differentiating spermatogonia, the mean lethal dose is 0.5 Gy.

Exposure to radioactivity has an adverse effect on the course of spermatogenesis in males. Irradiation of the male gonads with the dose of 3.5–6 Sv may lead to permanent infertility and an increase in the risk of congenital defects in offspring. In the case of lower doses, but higher than 150 mSv, temporary infertility may occur. In an experiment in which human sperm was exposed to X-rays, increasing the dose from the minimal to 80 Gy, and subsequently looking for DNA breakage in situ using DBD-FISH procedure, the minimum radiation dose causing DNA breakage was 30 Gy, which means that this was much higher than the dose received accidentally. When this dose was exceeded, the number of DNA single-strand breaks increased, and was positively correlated with radiation intensity [41,42,43].

Epidemiological studies concerning the effect of low doses of radiation on male fertility were carried out in the groups of males who, in association with occupational activity in X-ray units, were exposed to radioactive radiation. They were diagnosed with a decrease in sperm motility and reduction in the number of sperm with normal structure (the abnormalities mainly concerned the head), as well as intensification of vacuolization. In addition, in the sperm of these males, an intensification of DNA fragmentation and total methylation was observed. In the conducted study, the most negative effect on sperm morphology concerned gamma radiation. Similar damage to DNA structure and an increase in pathological forms was observed in the semen of males who participated in cleaning up the site of the Chernobyl nuclear power plant explosion.

Studies were also conducted concerning the effect of natural background radiation (NBR) on male fertility, focusing on its impact on sperm genetic material. The AZFc region was analyzed using DNA from the blood and sperm of 100 males living in the vicinity of the coastal peninsula in Kerala (India), who were exposed to NBR of elevated intensity in this region. These studies confirmed a mutagenic effect of natural radiation on chromosome Y in the context of its haploid status and clonal inheritance. The Y chromosome is inherited from father to son in an unaltered state; therefore, there is no possibility of recombination, and it is uniquely vulnerable to such mutations over generations [41,42,43].

## 12. Electromagnetic Fields

Electromagnetic radiation (EMR) originating from both the natural environment and human activity constantly affects living organisms. The effect of EMR on the reproductive system may occur as a result of the thermal effect, generation of oxidative stress, changes in the structure of proteins, and causing changes in ion transport through cell membranes; however, these pathomechanisms have not yet been fully explained. Based on studies conducted to date it is known that electromagnetic fields generate oxidative stress, disrupting the function of sperm mitochondria and causing the activation of cell membrane NADH oxidase [43].

Balance in the free radical system, which is affected by the electromagnetic fields, as well as the activity of ion channels, are together responsible for the process of sperm hyperactivation. Human spermatozoa, in order to become ready to fertilize an oocyte, undergo many metabolic changes affecting the cell membrane, which leads to capacitation occurring in the woman’s reproductive tract. As a result of these transformations, sperm can interact with the structures of the corona radiata, and subsequently with the zona pellucida of the oocyte. This process is necessary for fertilization. Possible premature capacitation taking place in the seminal plasma causes energy depletion of the sperm and reduces the chance for fertilization. Balance in the free radical system is the factor responsible for the proper moment of capacitation. Ion channels are responsible for the proper course of this process, including CatSper, a pH-regulated, calcium-selective ion channel, KSper (Slo3), and the voltage-gated channel Hv1. To date, it has been considered that many other channels regulate these processes; however, this was not supported by research, and only the implementation of the patch–clamp technique shed new light on the possibilities of opportunities to learn about this process. It is known that mutations and deletions of the genes responsible for the function of these channels are responsible for the existence of certain types of male infertility. The voltage-gated channel Hv1, which is an electrical voltage sensor responsible for processes related to fertilization, seems to be the most sensitive to the effect of EMF. The results of studies concerning the effect of EMF on the immune system cells confirmed that the frequency close to 15 Hz has the greatest impact on the transport of Ca2+ ions into the cell. Therefore, it may be expected that similar relationships will also concern sperm [43,44].

It has long been known that welding arcs are a very harmful source of EMF for males. Their radiation consists of intensive thermal radiation of high-temperature welding gases, welded or cut elements, electrode, and flux material, on which the lines and bands of radiation characteristic of these materials overlap. The gas burner flame temperature usually does not exceed 2000 K. Thus, this radiation consists mainly of infrared and light, and only hydrogen and acetylene burners are characterized by a higher combustion temperature and emit near-ultraviolet light. Scientific reports concerning the effect of welding on male fertility confirm that in males performing this profession, deterioration of the main sperm parameters occurs, and their partners are at an increased risk of miscarriage. The type of harmful effect on the male reproductive system depends on the type of materials to be welded and applies primarily to mild steel [43,44].

Exposure to strong EMF also concerns employees operating radar stations. Currently, various types of radars operate on frequencies from 3 MHz (HF band) to 110 GHz (W band). In the lower part of this range operate radars which measure the height of the ionosphere, and those using the phenomenon of reflection of electromagnetic waves from the ionosphere for detecting objects hidden beyond the horizon, often over a distance of thousands of kilometers. Most radars operate at frequencies ranging from several hundred megahertz to 100 GHz, including speed cameras known to drivers, which are used to take photos of cars and are based on the 34.3 ± 0.1 GHz band. The reports concerning their effect on male fertility are contradictory. Some researchers describe an increase in the percentage of abnormal sperm and reduction in sperm motility, with a simultaneous absence of effect on its density in employees of radar stations. In turn, other researchers describe a decreased sperm density in people exposed to radar waves. There are also reports that do not indicate any differences in the quality of sperm between males exposed to a radar, and those not exposed to its effect.

Due to the development of telecommunication technologies, at present, almost every person is exposed to EMF related to GSM. Our own study conducted at the beginning of the ‘era of mobile phones’ demonstrated an increase in the percentage of abnormal sperm and a decrease in the percentage of sperm in progressive movement, together with an increase in exposure to GSM. Studies concerning the effect of mobile phone technology on sperm have been continued by many researchers. They demonstrated that the waves emitted by mobile phones cause an increased production of the reactive oxygen species (ROS), leading to oxidative stress in sperm, and intensification of sperm DNA fragmentation. Similar relationships were confirmed on animal models. In a study in which mice were exposed to radiation of 900 mW/kg for 12 h daily for 7 days an adverse effect of EMR was observed on mitochondrial genome integrity. It was also proven that long-lasting telephone calls lead to an increase in temperature in the brain, which may affect the activity of the hypothalamic–pituitary–gonadal axis [43,44].

A significant exposure to electromagnetic fields also concerns males working on high-voltage electrical installations. It was found that among employees exposed for many years to contact with voltage of 400 kV, fewer children were born compared to the control group. Among children exposed to high voltage, the male gender prevailed. It was also observed that among offspring of males exposed to high voltage, congenital defects occurred more frequently.

There are also other scientific reports that confirm a beneficial effect of electromagnetic fields with lower frequency ranges on both sperm parameters and the function of tissues responsible for human reproduction. Some scientific studies confirmed that exposure of sperm to magnetic fields within the range from 10–50 Hz contributes to the improvement of the motility of human sperm, whereas others presented opposite results.

In animal models, while investigating the exposure of fish reproductive cells to magnetic fields, an improvement was observed in sperm motility parameters, and an increase in the percentage of fertilizations. Electromagnetic fields have found application in the treatment of prostate disorders in animals. A group of researchers confirmed that in dogs, the exposure of the prostate to frequencies of 4–12 Hz has a healing effect on prostate enlargement, not causing changes in sperm parameters of the animals. The results of studies confirming a beneficial effect of electromagnetic fields provide hope for their use in the treatment of human infertility; however, a long-term impact of this type of energy will require meticulous research, especially regarding possible epigenetic effects diagnosed only in future generations. Own studies of sperm exposed in vitro to weak EMF, close to that used at security checkpoints, showed an effect on DNA methylation [43,44].

At present, we are unable to specify what amount of energy related to EMF is harmful, neutral, or beneficial for human reproductive capacity. It may be expected that there are amounts of energy with neutral or beneficial effect, and only exceeding them may cause harmful effects.

## 13. Cigarettes

Cigarette smoking is among the main causes of morbidity and mortality worldwide. The relationship between cigarette smoking and reproductive capacity has been investigated for decades; however, there is a lack of prospective studies on a large scale that cover the whole population. Despite an increasing amount of evidence confirming the harmful effects of smoking, it still remains a common phenomenon, which is confirmed by the latest reports by the World Health Organization. More than one-third of all adult men worldwide use tobacco and nicotine [34,35].

The burden of the smoking habit contributes to the decline in the main parameters of basic and extended semen diagnostics. It has been proven that smoking results in a decrease in the level of zinc in sperm and reduces concentration, motility and percentage of sperm with normal structure, and intensifies DNA fragmentation. In heavy smokers, ultrastructural abnormalities are observed, which mainly concern microtubules and changes in the sperm tail. Nicotine smoking also impairs the acrosome reaction and capacitation, i.e., the processes ultimately necessary for fertilization. The harmful effect of smoking on reproductive processes is explained by the concept of oxidative stress; however, hypoxia resulting from smoking cigarettes may also be responsible for the impairment of the process of spermatogenesis [4,45].

There is also evidence suggesting that the adverse effect of smoking does not have to be due exclusively to toxins contained in cigarette smoke. A study assessing the effect of the sole nicotine on fertility in male rats showed that animals exposed to contact with this substance experienced a considerable reduction in motility and number of sperm. Thus, nicotine may also play an important role in unfavorable effect of smoking on fertility, irrespective of the toxins contained in smoke. Interestingly, sperm parameters under the effect of oral nicotine improved 30 days after cessation of its use, which suggests the element of reversibility of these effects [4,45].

Smoking may not only exert an adverse effect on sperm parameters in males but may also decrease the effectiveness of assisted reproductive techniques, such as in vitro fertilization (IVF) and intracytoplasmic sperm injection (ICSI). It is suggested that smoking by the father reduces the chances of achieving pregnancy as a result of in vitro fertilization procedures. In addition, smoking by the partner frequently causes an exposure of the mother to passive smoking, which may have further harmful effects for fertility of the woman.

Studies concerning the relationship between the level of testosterone and cigarette smoking in males are equivocal. Some studies suggest an elevated level of testosterone and dehydroepiandrosterone in semen in smokers, whereas others demonstrate that the mean levels of testosterone do not significantly differ between smokers and non-smokers [4,45].

Nevertheless, the majority of studies indicate that males suffering from infertility, or those who have difficulty becoming pregnant with their partner, should discontinue smoking to optimize their chances for successful conception.

Apart from the deterioration of the quality of sperm, smoking may also exert an effect on the state of health of the offspring. Embryos obtained from smokers may be of lower quality, and in children of smoking fathers, there occurs a higher risk of contracting cancer in later years. The pathophysiological mechanisms of this phenomenon have not yet been clearly recognized; however, it may be presumed that this is due to the intensification of oxidative stress, which is responsible for damage to DNA integrity [4,45].

## 14. Electronic Cigarettes

Electronic cigarettes are often considered as a safe substitute for the discontinuation of conventional cigarette smoking. The composition of the liquid applied in these devices is not always clearly specified and shows great differences according to the brand and manufacturer. Over 80 different chemical compounds were detected in liquids and aerosols. E-cigarettes contain nicotine, and the addition of flavorings considerably increases the toxicity of their fumes. The heat produced by the e-cigarette leads to the oxidation and decomposition of its components, ultimately creating harmful products in inhaled vapors. Although much lower levels of toxins have been found in electronic cigarette aerosols, compared to the smoke of a conventional cigarette, there are concerns about their potential impact on male and female fertility [46].

Most of the studies conducted to date were carried out in animal models, whereas those concerning humans are scarce. However, the results observed in animal models suggest that care should be taken using electronic cigarettes, and it is necessary to conduct a larger amount of research in order to identify the potential adverse effect of this stimulant on fertility. The widespread use of these devices is alarming, and people should be warned against the effects of stimulants on reproductive health [46].

Due to the numerous ingredients and different concentrations of substances contained in the electronic liquid, it is difficult to specify the precise toxic effect of e-cigarettes on fertility because each component separately may have a harmful effect. The frequent use of e-cigarettes disrupts the hypothalamic–pituitary axis, causing changes in gonadal function and sperm quality. Researchers confirmed that in male rats exposed to fumes of electronic cigarettes, there occurred an increased apoptosis in spermatogonia and spermatocytes, changes in the morphology and function of the seminiferous epithelium, as well as developmental defects. Other studies connected the use of e-cigarettes with steroidogenesis disorders and global testicular disorganization, accompanied by considerable exfoliation of reproductive cells. In the context of exposure to electronic cigarettes, a low testicular mass and a higher number of apoptotic cells in the testes was observed. The sperm of rats exposed to fumes of these devices showed an increased teratozoospermia affecting especially the flagella. Investigations also demonstrated that exposure to e-cigarettes may also exert an effect on sperm chromatin integrity [46,47].

Summing up, taking into account the many potential harmful effects of e-cigarettes described on animal model, it may be considered that smoking e-cigarettes is not a safe alternative for conventional smoking. Scientific evidence concerning the effect of the use of these devices is alarming, and people trying to achieve pregnancy should be aware of the potential effect of electronic cigarettes on their health.

## 15. Alcohol

Studies suggest that the chronic and excessive consumption of alcohol has an adverse effect on the secretion of male sex hormones and the quality of sperm. On the other hand, the impact of the moderate consumption of ethanol is still under discussion.

The everyday consumption of alcohol by males causes a reduction in the volume of semen, density, and percentage of sperm with normal structure, and sperm motility. In alcoholics, there occurs a disturbance in the function of the hypothalamic-pituitary-testicular axis resulting in a decrease in the level of testosterone. In persons excessively consuming this stimulant, a partial or complete arrest of spermatogenesis and reduction in the mean weight of the testes may be observed [4,45].

Experiments in vitro showed acrosome dysfunction occurring during capacitation of human and animal sperm incubated in ethanol, which reduces their fertilization capacity. This is probably due to the ability of ethanol to change lipid fluidity and membrane permeability through oxidation of lipids and cell membrane proteins. In rats, after exposure to ethanol a decreased sperm motility, changes in meiotic divisions, reduced gamete viability, and a larger number of sperm with poorly condensed chromatin were observed [4,45].

This effect was not noted in males who consumed alcohol occasionally. The researchers observed even better sperm motility in persons drinking sporadically, compared to those who did not consume alcohol at all. In fact, the relationship between the quality of sperm and the amount of alcohol consumed still remains controversial [4,45].

Apart from the fact that it is an important public and social problem, alcohol consumption may also considerably affect male reproduction. Many studies, both on humans and animals, confirmed a relationship between the chronic consumption of ethanol and poor quality of sperm, mainly due to an excessive production of ROS as a result of EtOH metabolism. By acting as genotoxic agents, EtOH and its metabolites change the expression of specific genes involved in the hormonal regulation of spermatogenesis and increase sperm DNA fragmentation, potentially with transgenerational effects on offspring. Despite the role alcohol plays in contributing to male infertility, the amount of this substance clearly harmful to the reproductive function in males has not yet been determined [4,45].

## 16. Summary

The presented current state of knowledge concerning the risks created by the selected environmental factors may be approached as an introduction to this scope of problem. At present, we are unable to identify all the environmental factors, the interactions between them, and their full effect on male fertility.

It is known that male fertility is only one of the factors influencing birth rate. The spread of male factor infertility varies by geographic region and varies in other parts of the world [48]. A similar situation applies to exposure to environmental factors [49]. In recent years, the demographic situation of developed countries has been disrupted by the COVID pandemic and then by spreading war conflicts. These phenomena contributed to postponing the decision to have children. On the other hand, the economic situation, climate change and war conflicts have contributed to the intensification of migration from countries with a better demographic situation to countries with low natural growth. The war crisis in Ukraine has caused an inflow of lower quality agricultural products (more contaminated with plant protection products not used in the European Union) to neighboring countries. The coming years will show what the consequences of these phenomena will be.

The main weakness of many of the presented research results is the lack of possibility of isolating most simultaneously operating factors from each other. Additionally, the issue of interactions between individual environmental factors has not been fully recognized. The investigation of the effect on male fertility is also hindered by the fact that the process of spermatogenesis lasts for about 3 months, and we do not know at what point in its duration the sperm is most exposed to damage by external factors. In addition, standard analysis of semen cannot fully assess fertilization ability of sperm, and consequently assess a man’s fertility.

Despite difficulties related to the methodology of the conducted study, the results published to date should evoke great concerns about the quality of the environment in which we live, as well as a concern about the effect of environmental factors not only on male fertility, but on all aspects of human health. Most of environmental pollutants affect the male body by causing oxidative stress and exert an effect on the endocrine system. This disrupts the function of most tissues and has an impact on the genetic material and epigenetic processes. This creates the need for further research concerning this scope of problems in the future, while the knowledge available today should build awareness of avoiding threats resulting from exposure to environmental factors.

The current state of knowledge about the threats caused by environmental factors should motivate legislators to implement legal solutions protecting against their effects on health. Moreover, this raises the need to intensify the education of children and adolescents in order to reduce exposure to environmental factors [50]. Significant environmental pollution creates the need to look for solutions and means of protection against it. One potential solution is pharmacological protection [16]. Due to the existence of unexplored areas of knowledge in this field, long-term, interdisciplinary research is needed to create strategies to mitigate the effects of exposure to environmental factors. Undoubtedly, such actions would require coordination by a group of independent experts in this field. Potential changes in economic strategies aimed at protecting against harmful environmental factors may involve high financial costs, but in the future, it may reduce expenditure on healthcare. It should be emphasized that scientists, politicians and representatives of all branches of industry and agriculture are jointly responsible for the current and future health of the human population (including reproductive health). These communities should urgently get involved in establishing new ecological criteria.

## Data Availability

Not applicable.
